# Impact of the COVID‐19 pandemic on paediatric renal tumour presentation and management, a SIOP renal tumour study group study

**DOI:** 10.1002/cam4.6358

**Published:** 2023-07-26

**Authors:** Prakriti Roy, Sophie E. van Peer, Rana Dandis, Catriona Duncan, Joaquim Caetano de Aguirre‐Neto, Arnauld Verschuur, Beatriz de Camargo, Henrike E. Karim‐Kos, Luna Boschetti, Filippo Spreafico, Gema L. Ramirez‐Villar, Norbert Graf, Harm van Tinteren, Kathy Pritchard‐Jones, Marry M. van den Heuvel‐Eibrink

**Affiliations:** ^1^ Princess Máxima Center for Pediatric Oncology Utrecht The Netherlands; ^2^ Great Ormond Street Hospital for Children London UK; ^3^ Paediatric Haemato‐oncology Hospital Santa Casa de Belo Horizonte Belo Horizonte Brazil; ^4^ Department of Paediatric Oncology & Haematology La Timone Children's Hospital Marseille France; ^5^ Grupo Brasileiro de Tumores Renais (Brazilian Renal Tumor Group) São Paulo Brazil; ^6^ Department of Research Netherlands Comprehensive Cancer Organisation (IKNL) Utrecht The Netherlands; ^7^ Department of Medical Oncology and Hematology, Pediatric Oncology Unit Fondazione IRCCS Istituto Nazionale dei Tumori di Milan Milan Italy; ^8^ Department of Paediatric Oncology Hospital Universitario Virgen del Rocío Seville Spain; ^9^ Department of Paediatric Oncology & Haematology Saarland University Homburg Germany; ^10^ UCL Great Ormond Street Institute of Child Health, University College London London UK; ^11^ Division of Child Health, Wilhelmina Children's Hospital University Medical Center Utrecht The Netherlands

**Keywords:** childhood cancer, COVID‐19 pandemic, global pandemic, nephroblastoma, SARS‐CoV‐2‐infection, Wilms tumour

## Abstract

**Background:**

The COVID‐19 pandemic had global catastrophic effects on the management of non‐communicable diseases including paediatric cancers. Restrictions during the start of 2020 complicated timely referrals of patients to specialized centres. We aimed to evaluate the pandemic’s impact on the number of new diagnoses, disease characteristics and management delay for paediatric renal tumour patients included in the SIOP‐RTSG‐UMBRELLA study, as compared with data from a historical SIOP‐RTSG trial (2005–2009).

**Methods:**

The number of intensive care admissions, population mobility rates and national lockdown periods/restrictions were used as proxies of the pandemic’s severity and impact on societies. Clinical and tumour data were extracted from the SIOP‐RTSG‐UMBRELLA study and from historical SIOP‐RTSG trials.

**Results:**

During the first lockdown in Europe, the number of newly diagnosed patients decreased following restrictions and population immobilisation. Additionally, there was a higher proportion of advanced disease (37% vs. 17% before and after COVID‐9, *p* < 0.001) and larger median tumour volume (559 cm^3^ vs. 328 and 434 cm^3^ before and after, *p* < 0.0001). Also in Brazil, the proportion of advanced disease was higher during the national decrease in mobilisation and start of restrictions (50% and 24% vs. 11% and 18% before and after, *p* < 0.01). Tumour volume in Brazil was also higher during the first months of COVID‐19 (599 cm^3^ vs. 459 and 514 cm^3^), although not significant (*p* = 0.17). We did not observe any delays in referral time nor in time to start treatment, even though COVID‐19 restrictions may have caused children to reach care later.

**Conclusion:**

The COVID‐19 pandemic briefly changed the tumour characteristics of children presenting with renal tumours. The longer‐term impact on clinical outcomes will be kept under review.

## Graphical abstract

The COVID‐19 pandemic briefly changed the tumour characteristics of children presenting with renal tumours. During the first peak of COVID‐19, more children presented with metastatic disease and median tumour volume was higher. Further analyses after longer follow‐up is needed to study the influence on clinical outcomes.

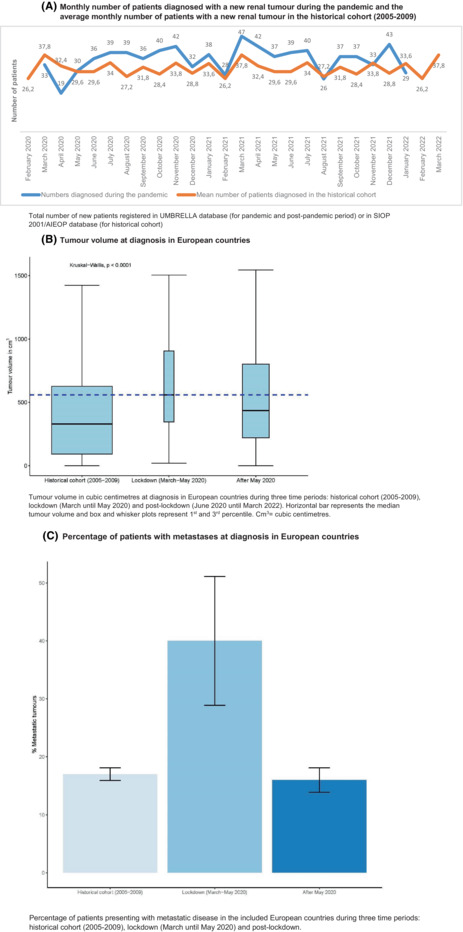

## 1 INTRODUCTION

COVID‐19 was declared a pandemic by the World Health Organization (WHO) in the first months of 2020.^1^ In the beginning of March 2020, most countries in Europe imposed the first lockdown and other social restriction measures at a national level, the aims being to slow down infection transmission, to reduce the burden on healthcare systems and to prevent subsequent mortality.^2^ This had already been proven to be effective to some extent in China in the preceding months and thereafter it was adopted on a global scale.^3^ It was anticipated that without such restrictions there would be more hospital admissions and deaths due to SARS‐CoV‐2 infections.^4^

It was also anticipated that these unprecedented global restrictions could have a potential negative impact on the management of non‐communicable diseases such as cancer. This was reflected in a WHO report, which proposed adoption of alternative strategies to handle the overburdened health care system.^5^ As a response to this challenge, the global paediatric oncology groups published a collaborative guideline to support healthcare providers to guarantee safe and effective care for children with cancer. This guideline focusses on the six index cancer types highlighted in the WHO Global Initiative for Childhood Cancer (GICC), which includes Wilms tumour.^6^

Despite these efforts, early reports from many countries and regions mentioned lower cancer referral rates in adults and children in the first phase of the pandemic. This observation was attributed to fear of SARS‐CoV‐2 infection and simultaneous national lockdowns or restrictions, reducing access to medical services.^7^, ^8^ This resulted in delayed diagnoses^7^, ^9^, ^10^, ^11^ and inadequate access to treatment resources. Also, elective postponement of surgery due to shortages of staff and admission capacity (including limited post‐operative pediatric intensive care unit [PICU] capacity) induced delay.^12^, ^13^ These adverse impacts varied not only by country or region, but also by type of cancer.^10^, ^14^

One of the keys to excellent survival for most cancers is early diagnosis and management, especially for childhood cancers, which are typically highly proliferative cancer types.^15^, ^16^, ^17^ This prompt diagnosis and management was feared to be restricted during the pandemic. Several model‐based reports showed prediction of an excess of population based deaths or adverse outcomes in cancer patients (in all age categories) due to diagnostic delays.^18^, ^19^ So far, studies in both adults and children have reported a decrease in the number of patients diagnosed during the period of pandemic. Moreover, an increased number of advanced disease was observed in some adult cancers.^20^, ^21^ While most reports on COVID‐19 infections in paediatric cancer patients did not show a more severe course of the infection,^22^, ^23^, ^24^, ^25^ serious indirect consequences of the pandemic have been described in some paediatric cancer patients.^26^, ^27^, ^28^ The latter was especially the case in low and middle income countries, where delay and shortage of material and personnel played a larger role than in high income countries.^28^

The aforementioned reports reflect mostly either adult cancer and/or national cancer registry perspectives. Therefore, the possibility to ascertain the impact on individual cancer types and organisation of care for subsets of patients on a global scale is still limited.^23^, ^29^, ^30^, ^31^, ^32^ The paediatric oncology setting in general is different with respect to referral speed, based on the need for rapid management of the most highly proliferating cancer types in childhood. Therefore, our aim is to report on the impact of the first phase of the pandemic on time to diagnosis, presenting symptoms, tumour characteristics (volume and stage), and potential delay in management of paediatric renal tumour patients registered in the International Society of Paediatric Oncology Renal Tumour Study Group (SIOP‐RTSG) UMBRELLA prospective clinical study.^33^

## 2 MATERIALS AND METHODS

From June 2019 on, in Europe, Asia and South America, children with a renal tumour could be registered in the SIOP‐RTSG‐2016 UMBRELLA protocol (further referred to as UMBRELLA). This study provides standard of care guidelines for treatment, diagnostics (including central review) and biobanking. Currently, over 25 countries in Europe, Asia and South America are participants in UMBRELLA.

To assess severity of the COVID‐19 pandemic and the impact on societies, restrictions periods, cumulative monthly intensive care unit (ICU) admissions, and general population mobility data (Institute of Health Metrics and Evaluation [IHME] https://www.healthdata.org/covid) were used as proxies.^34^ Numbers of daily reported new COVID‐19 cases were not considered representative, as test settings were insufficient and not comparable in the first pandemic phase.

To study the influence of COVID‐19, we analysed Wilms tumour characteristics (volume and stage), time to diagnosis (defined by the date at which a renal tumour was confirmed on radiology) and to start of treatment, route to diagnosis and presenting symptoms. Inclusion criteria for countries to participate in this study were that they had initiated UMBRELLA^33^ and registered at least 30 patients in the indicated period. In addition, historical data of five consecutive years (2005–2009), registered in the previous SIOP WT 2001 trial (or the AIEOP [Associazione Italiana Ematologica Oncologica Pediatrica] for Italy), had to be available as a reference. Also, national epidemiological data were necessary regarding the course of the COVID‐19 pandemic. This included data on COVID‐19 restrictions, daily ICU admission occupancy and national mobility data (IHME). Patients with active COVID‐19 were not excluded from the analyses.

Date of diagnosis (the date that the first imaging modality confirmed a renal tumour) was used to calculate the number of newly diagnosed patients with a renal tumour per month, since study registration date may be later. We performed the assessment of tumour volume at diagnosis, tumour stage, and treatment delay, for every country separately and patient data were extracted from the study database.

Only patients with Wilms tumours were included in the analyses of tumour volume and amount of patients with metastases. Patients were excluded from these analyses when they had bilateral disease or when information on centrally reviewed radiology and/or pathology was missing.

These data were compared with historical SIOP WT 2001 trial data from 2005 to 2009.^33^, ^35^ Tumour volumes were measured by abdominal magnetic resonance imaging (MRI), computed tomography (CT) scan or, if not available, ultrasound (US). The median three‐dimensional volumes at diagnosis of the largest lesion (in case of multifocal tumours) were calculated in cm^3^ (volume = 0.523 times the three dimensions of the tumour). To compare patients with metastases during COVID‐19 to the historical cohort, we used percentages relative to the total number of stage I–IV disease patients.

Presenting symptoms were collected in the four pre‐defined categories that are indicated on the UMBRELLA study registration form (tumour specific symptoms, non‐specific assessment, screening because of a known genetic predisposition or routine check‐up).The category tumour‐specific symptoms in the COVID‐19 period was quantified as a percentage of the total group and compared with the period before and after COVID‐19. Data on route to presentation were not included in the previous SIOP‐RTSG trial (SIOP WT 2001), hence these data are only available from April 2019 onwards.

Referral time was defined as the number of days from the start of symptoms to the date of diagnosis, and time to treatment as the number of days from diagnosis to the start of any treatment. Time to surgery for patients who received pre‐operative chemotherapy was defined as the number of days from the start of chemotherapy to the surgery and for patients who underwent upfront surgery, as the number of days from diagnosis to the surgery. The time to surgery was analysed separately for localised and metastatic disease, as patients receive 4 or 6 weeks of pre‐operative chemotherapy, respectively.^33^ This was compared with the recommended surgery timing in the UMBRELLA protocol. Patients with bilateral disease (stage V) were not included as this group of patients is small and the treatment approach varies significantly due to the complexity of surgical planning. All these variables were analysed for individual months during the pandemic. Time to treatment could not be compared with the historical data because it was not registered in the period of 2005–2009.

The data from European countries were merged, since the countries had a comparable course of the pandemic regarding restriction periods and ICU admissions. The Brazilian data were evaluated separately, as it was assumed that restrictions and peak of COVID‐19 pandemic occurred later than in Europe due to lack of a uniform, nation‐wide governmental lockdown and due to seasonal differences.

For the current study, we focussed on the very first period of the pandemic, as procedures such as testing and sequential vaccination strategies were implemented later with wide variation across the included countries.

Statistical analysis was performed using a Kruskal–Wallis test to investigate the impact of the lockdown on the observed tumour volume. In Europe, we analysed this during three periods: overall historical cohort (2005–2009), during the first lockdown (March until May 2020) and the subsequent period (until 31 March 2022). For Brazil, the COVID‐19 social distancing restrictions were not all initiated at the same time, as it is a large and heterogeneous country. Therefore, we used population mobility tracked by mobile phone as an additional tool to assess the course of the pandemic. Due to seasonal differences, we expected to observe the effects of the pandemic 2 months later than in Europe. Therefore, we split the periods for Brazil into the period of initiation of measures and lockdowns in Europe (March and April 2020), the period of most restrictions in the largest parts of Brazil (May until July 2020)^36^, ^37^ and thereafter (until 31 March 2022).

A pairwise comparison using Dunn's test and Bonferroni correction for multiple testing was performed to see if the periods had different effects. The Chi‐Square Test of Independence was used to compare the percentage of metastatic patients between the various periods described above. The statistical analyses were performed using R software version 4.2.1.

## 3 RESULTS

### 3.1 Course of the first phase of the COVID‐19 pandemic in Europe and Brazil

**FIGURE 1:**
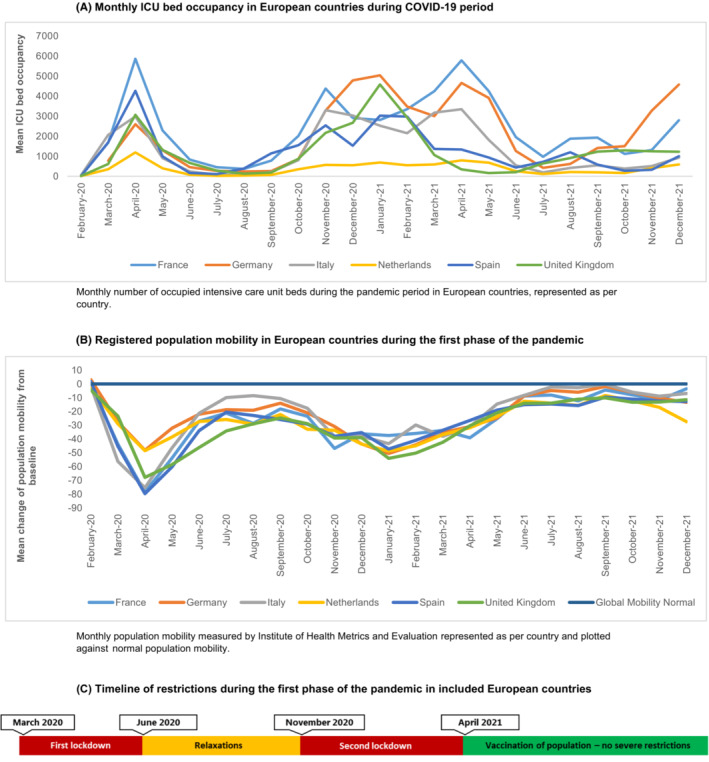
Epidemiological data and timeline of restrictions during the first phase of the COVID‐19 pandemic in Europe. (A) Monthly ICU bed occupancy in European countries during COVID‐19 period. Monthly number of occupied intensive care unit beds during the pandemic period in European countries, represented as per country. (B) Registered population mobility in European countries during the first phase of the pandemic. Monthly population mobility measured by Institute of Health Metrics and Evaluation represented as per country and plotted against normal population mobility. (C) Timeline of restrictions during the first phase of the pandemic in included European countries.

**FIGURE 2:**
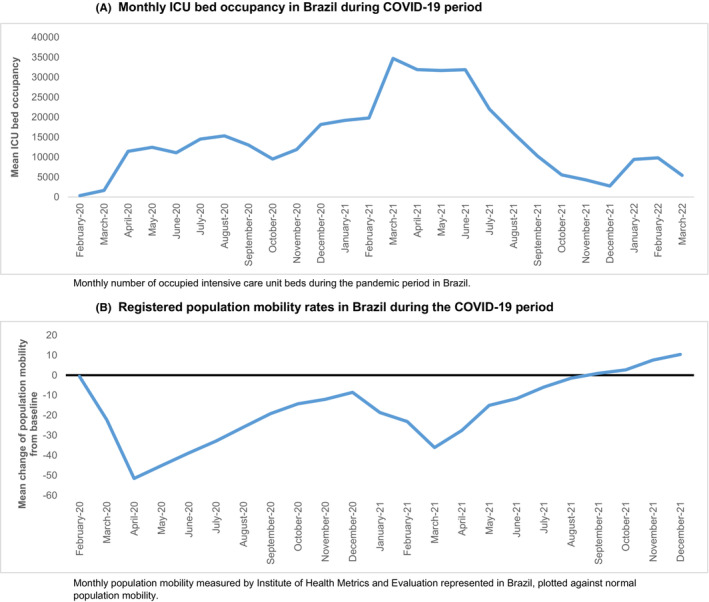
Epidemiological data during the first phase of the COVID‐19 pandemic in Brazil. (A) Monthly ICU bed occupancy in Brazil during COVID‐19 period. Monthly number of occupied intensive care unit beds during the pandemic period in Brazil. (B) Registered population mobility rates in Brazil during the COVID‐19 period. Monthly population mobility measured by Institute of Health Metrics and Evaluation represented in Brazil, plotted against normal population mobility.

Included countries were Germany, France, Italy, the Netherlands, Spain, United Kingdom and Brazil. ICU admission numbers (Figures 1A and 2A), global population mobility data (Figures 1B and 2B) and the lockdown periods (Figure 1C) showed that the burden of the pandemic in Europe reached its peak in the middle of March and lasted until the end of May of the year 2020. From the beginning of June 2020, some measures were relaxed based on reduced burden in the hospitals in most countries. In Europe, an immediate decrease in population mobility followed the restrictions, and mobility slowly increased again in June 2020 (Figure 1B). Second peaks of the pandemic were observed in the months October 2020 until April 2021, at the end of which most countries had implemented national testing strategies and vaccination programs for the general population.

In Brazil, ICU admissions increased slightly later than in Europe, from April 2020 onwards and reached a second peak in March until June 2021 (Figure 2A). Population mobility was decreasing from March 2020 onwards, together with the start of the social distancing advice in most regions, showing a superimposable pattern to that observed in Europe (Figure 2B). A second period of COVID‐19 restrictions occurred in March 2021 following a peak in ICU admissions, however, we observed large regional variations due to the heterogeneity and size of the country.^36^, ^37^

### 3.2 Number of patients with a newly diagnosed renal tumour during the first phase of the pandemic

**FIGURE 3:**
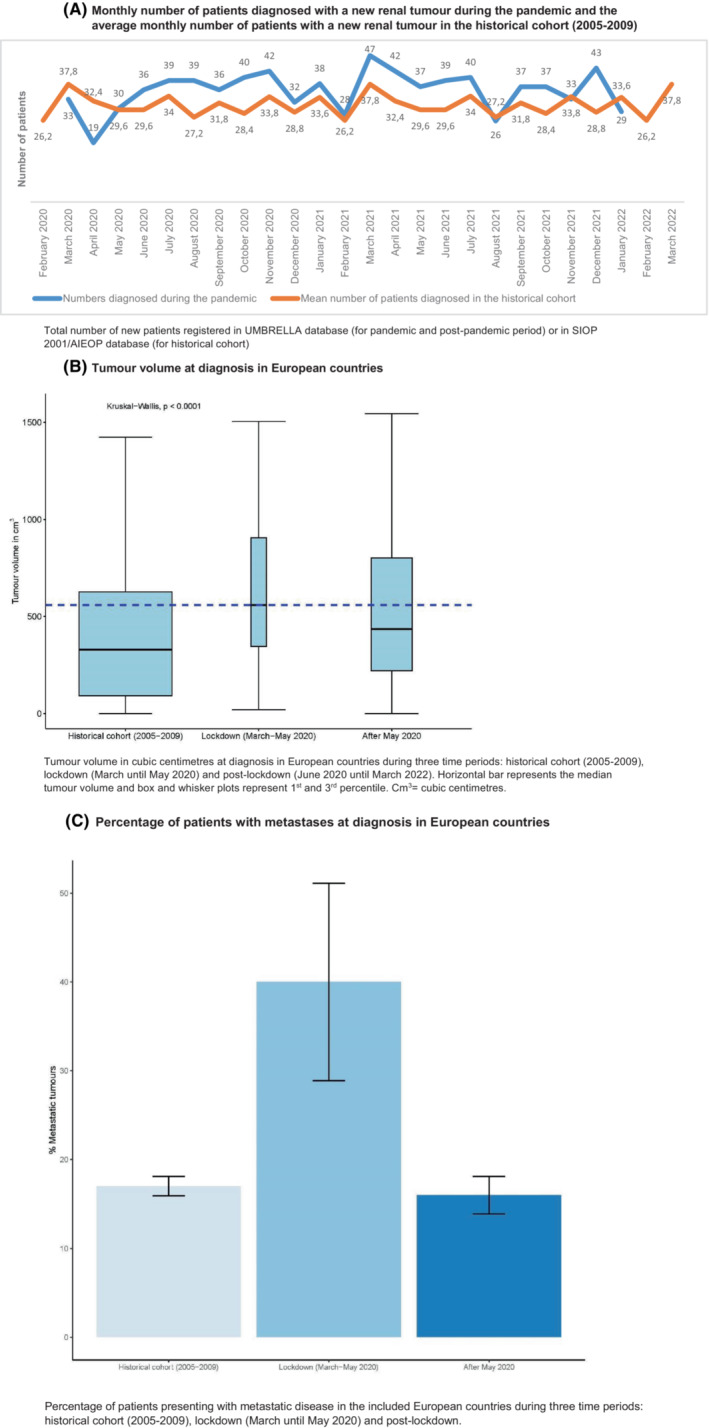
Newly registered patients, tumour volume and disease stage during the first phase of the pandemic in Europe. (A) Monthly number of patients diagnosed with a new renal tumour during the pandemic and the average monthly number of patients with a new renal tumour in the historical cohort (2005–2009). Total number of new patients registered in UMBRELLA database (for pandemic and post‐pandemic period) or in SIOP 2001/AIEOP database (for historical cohort). (B) Tumour volume at diagnosis in European countries. Tumour volume in cubic centimetres at diagnosis in European countries during three time periods: historical cohort (2005–2009), lockdown (March until May 2020) and post‐lockdown (June 2020 until March 2022). Horizontal bar represents the median tumour volume and box and whisker plots represent 1st and 3rd percentile. Cm3 = cubic centimetres. (C) Percentage of patients with metastases at diagnosis in European countries. Percentage of patients presenting with metastatic disease in the included European countries during three time periods: historical cohort (2005–2009), lockdown (March until May 2020) and post‐lockdown.

The number of patients registered with a newly diagnosed renal tumour in these European countries is depicted in Figure 3A. In March 2020, 33 patients with a renal tumour were registered in UMBRELLA, compared to a monthly average of 38 patients in the historical cohort (i.e. 2005–2009). In April 2020, 19 new renal tumour patients were registered, versus an average of 32 patients in the historical cohort (Figure 3A). In the months following the first lockdown period (i.e. after May 2020), a higher number of newly diagnosed patients were registered compared to the lockdown period and the historical cohort.

**FIGURE 4:**
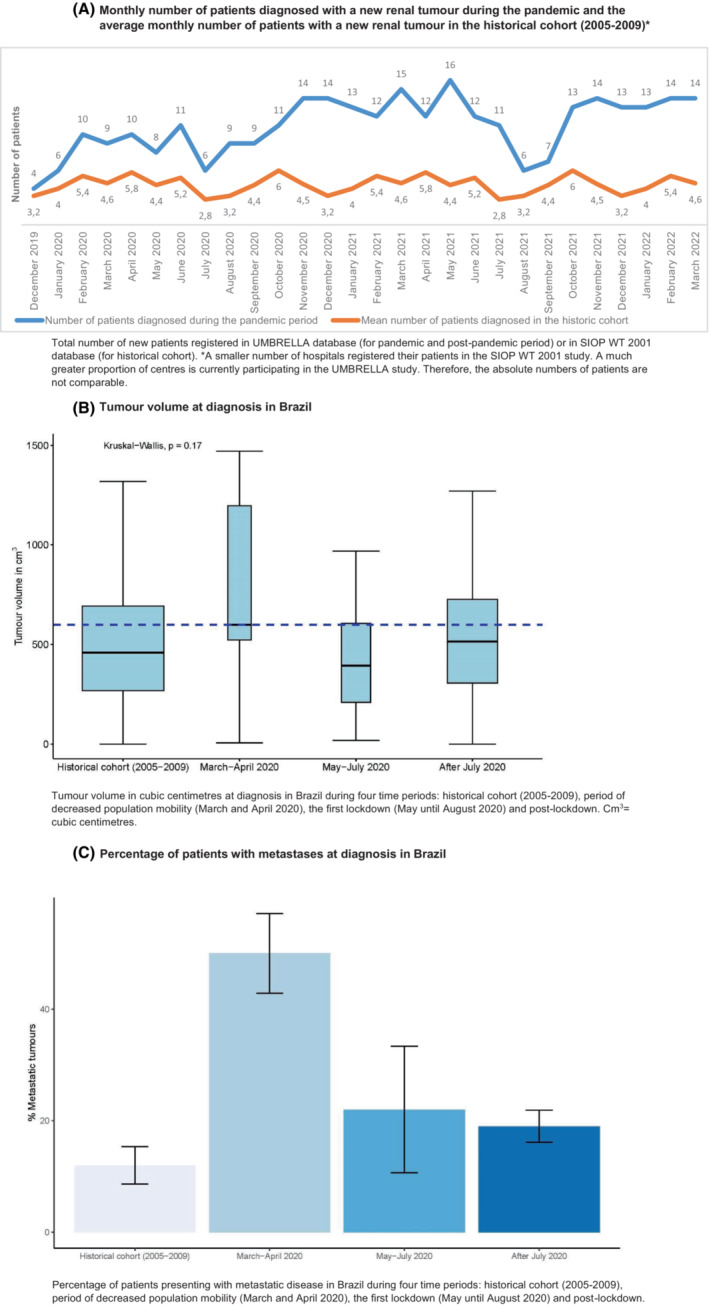
Newly registered patients, tumour volume and disease stage during the first phase of the pandemic in Brazil. (A) Monthly number of patients diagnosed with a new renal tumour during the pandemic and the average monthly number of patients with a new renal tumour in the historical cohort (2005–2009)*. Total number of new patients registered in UMBRELLA database (for pandemic and post‐pandemic period) or in SIOP WT 2001 database (for historical cohort). *A smaller number of hospitals registered their patients in the SIOP WT 2001 study. A much greater proportion of centres is currently participating in the UMBRELLA study. Therefore, the absolute numbers of patients are not comparable. (B) Tumour volume at diagnosis in Brazil. Tumour volume in cubic centimetres at diagnosis in Brazil during four time periods: historical cohort (2005–2009), period of decreased population mobility (March and April 2020), the first lockdown (May until August 2020) and post‐lockdown. Cm^3^ = cubic centimetres. (C) Percentage of patients with metastases at diagnosis in Brazil. Percentage of patients presenting with metastatic disease in Brazil during four time periods: historical cohort (2005–2009), period of decreased population mobility (March and April 2020), the first lockdown (May until August 2020) and post‐lockdown.

In Brazil, in the period of decreased population mobility and start of social distancing measures in most regions, in March and April 2020, 9 and 10 patients were registered, respectively. In May, June and July 2020, 8, 11 and 6 patients were registered, respectively (Figure 4A). Again, a slightly higher number of patients was included in the months thereafter. A direct comparison of total registrations with the historical cohort was not possible for Brazil due to a lower number of participating centres in the period 2005–2009 in SIOP WT 2001 than in the UMBRELLA study.

### 3.3 Wilms tumour volume at presentation during the pandemic

In Europe, the median Wilms tumour volume was 559 cm^3^ at diagnosis during the first lockdown period (March until May 2020), compared to 328 cm^3^ in the historical cohort (*p* < 0.0001). The median tumour volume after the lockdown period was 434 cm^3^ (*p* = 0.17) (Figure 3B, Table 1).

**TABLE 1:** Tumour volume at diagnosis in European countries and Brazil before and during the COVID‐19 pandemic.

	Historical cohort (2005–2009) (*N* = 1603)	March–May 2020 (*N* = 43)	After May 2020 (*N* = 309)	–	*p*‐value
Europe					
Median volume (cm^3^)	328.03	559.19	434.49	–	<0.001*
	Historical cohort (2005–2009) (*N* = 159)	March–April 2020 (*N* = 13)	May–July 2020 (*N* = 20)	After July 2020 (*N* = 60)	
Brazil					
Median volume (cm^3^)	459.03	598.79	394.47	513.85	0.13

* *p* < 0.001 for the comparison between the period of March and May 2020 and the historical cohort and for the comparison of the period after May 2020 and the historical cohort.

In Brazil, the median tumour volume from May until July 2020, was 394 cm^3^ compared to 459 cm^3^ in the historical cohort (2005–2009). In the period with decreased population mobility with the start of the first COVID‐19 measures (March and April 2020), the median tumour volume was 599 cm^3^. After July 2020, the median tumour volume was 514 cm^3^. The differences in volume between these four periods was not significant (*p* = 0.17) (Figure 4B, Table 1).

### 3.4 Disease stage of Wilms tumour patients at diagnosis

During the first lockdown in Europe (March until May 2020), 22 of 59 patients (37%) had metastatic disease compared to 261/1538 (17%) in the historical cohort and 78/460 (17%) in the period directly after the first lockdown (*p* < 0.001) (Figure 3C, Table 2).

**TABLE 2:** Percentage of patients with metastatic tumours in European countries before and during the COVID‐19 pandemic.

	Historical cohort (2005–2009) (*N* = 1538)	March–May 2020 (*N* = 59)	After May 2020 (*N* = 460)	–	*p*‐value
Europe					
Localised	1277 (83.0%)	37 (62.7%)	382 (83.0%)	–	<0.001
Metastasized	261 (17.0%)	22 (37.3%)	78 (17.0%)	–	
	Historical cohort (2005–2009) (*N* = 191)	March–April 2020 (*N* = 14)	May–July 2020 (*N* = 21)	After July 2020 (*N* = 121)	
Brazil					
Localised	170 (89.0%)	7 (50.0%)	16 (76.2%)	99 (81.8%)	0.00184
Metastasized	21 (11.0%)	7 (50.0%)	5 (23.8%)	22 (18.2%)	

In Brazil, 5/21 patients (24%) had metastatic disease in the period from May until July 2020, in the historical cohort 21/191 (11%), and directly after July 2020 these were 22/121 patients (18%). In March and April 2020 in which the population mobility started decreasing (March and April 2020), 7/14 patients (50%) had metastatic disease, which was significantly higher compared to all three other periods (*p* < 0.01) (Figure 4C, Table 2).

### 3.5 Route to diagnosis and presenting symptoms

Pre‐, during and post‐lockdown, 63%, 63% and 61% of patients presented with tumour specific symptoms in Europe, respectively. In Brazil, this was assessed for the pre‐COVID‐19 period, March and April 2020, May until July 2020 and the period thereafter. In these periods, 87%, 95%, 92% and 85% of patients presented with tumour specific symptoms, respectively (Table S1).

Diagnosis following assessment of non‐specific symptoms was 22%, 22% and 25% in the three periods in Europe, respectively. In Brazil, this was 11%, 5%, 0% and 15% for the aforementioned four periods, respectively. For diagnosis via screening for a known renal tumour predisposition, these percentages were 13%, 4% and 15% in Europe and 3%, 0%, 8% and 0% for Brazil. Discovery of a Wilms tumour during routine child health checks was the route to diagnosis in 9%, 12% and 11% of patients in Europe. In Brazil, no child with Wilms tumour was diagnosed through this route (Table S1).

Details of presenting symptoms as reported by the parent or patient are specified for three (Europe) or four (Brazil) time periods in Table S2 and Figures S2 and S3.

### 3.6 Time to diagnosis

In Europe, during the first lockdown, the median number of days from first symptoms to diagnosis was 2 days (IQR 1–6 days). In Brazil, during the period of the most severe COVID‐19 restrictions (May until July 2020) this was 6 days (IQR 3–37 days). There are no data from historical cohorts available to compare the duration from symptoms to diagnosis (Table 3).

**TABLE 3:** Time to diagnosis and time to treatment during the first period of the pandemic.

In Europe	Number of days (IQR)	In Brazil	Number of days (IQR)
Time to diagnosis		Time to diagnosis	
February 2020	5 (1–7)	February 2020	8 (4.75–15.75)
March 2020	4 (1–6)	March 2020	8 (4.5–17.25)
April 2020	2 (1–4)	April 2020	8.5 (2–22)
May 2020	1 (0–3)	May 2020	3.5 (1–21.3)
June 2020	2 (0–5)	June 2020	6 (4.5–13)
July 2020	1.5 (0–4.5)	July 2020	52 (6.8–116.8)
Median for lockdown 1 (March–May 2020)	2 (1–5.5)	Median for period of most severe restrictions (May–July 2020)	6 (3–37)
Time to any treatment		Time to any treatment	
February 2020	7 (5.5–10.75)	February 2020	6.5 (4.25–19.75)
March 2020	6.5 (4–7.75)	March 2020	4 (1.75–7)
April 2020	7 (3–8)	April 2020	4 (1–7)
May 2020	5 (2–7)	May 2020	6 (1.75–16.5)
June 2020	6 (2.75–7.25)	June 2020	6.5 (1.5–7.75)
July 2020	4.5 (2.25–7)	July 2020	4.5 (3.25–12.5)
Median for lockdown 1 (March–May 2020)	6 (2–7.75)	Median for period of most severe restrictions (May–July 2020)	5 (1–16.5)
Time to surgery after pre‐operative chemotherapy for localised Wilms tumour		Time to surgery after pre‐operative chemotherapy for localised Wilms tumour	
February 2020	36 (35–37)	February 2020	33 (28.5–34)
March 2020	43 (28.5–49)	March 2020	33 (32–34)
April 2020	34.5 (33.25–37.25)	April 2020	34 (29–39)
May 2020	30 (29.25–31‐75)	May 2020	33 (28–34)
June 2020	32.5 (31.75–34.25)	June 2020	33.5 (31.5–34.75)
July 2020	32.5 (27.5–36)	July 2020	30.5 (25.25–32.75)
Time to surgery after pre‐operative chemotherapy for metastatic Wilms tumour		Time to surgery after pre‐operative chemotherapy for metastatic Wilms tumour	
February 2020	50 (47.5–52.5)	February 2020	47
March 2020	49.5 (43.25–52.75)	March 2020	45.5 (38–47.25)
April 2020	44.5 (44–48.75)	April 2020	44 (32–52.25)
May 2020	42 (42–42)	May 2020	69.5 (69.25–69.75)
June 2020	47 (47–47.5)	June 2020	48 (43.5–52)
July 2020	49 (42–81)	July 2020	No patients with metastatic Wilms tumour in this month

Abbreviation: IQR, interquartile range.

### 3.7 Time to initial treatment (diagnosis to pre‐operative chemotherapy)

In the 3 months during the first lockdown in Europe (March until May 2020) the median duration to any type of treatment (chemotherapy or upfront surgery) was 6 days (IQR 2–8 days), and in Brazil (from May until July 2020) 5 days (IQR 1–17 days) (Table 3). There are no data from historical cohorts available to compare the time to treatment to, but in general, this time should be as short as possible (Table 3).

### 3.8 Time to treatment (pre‐operative chemotherapy to surgery)

According to the UMBRELLA protocol, surgery preferably takes place within 28–35 days for localised tumours and within 42–49 days for patients with metastatic disease after start of pre‐operative chemotherapy. Median time to surgery in Europe during the first 3 months of the lockdown (March, April, May) was 43, 35 and 30 days for patients with localised disease and 50, 45 and 42 days for patients with metastatic disease, respectively. In Brazil, during the months May until July 2020 the median time from start of pre‐operative chemotherapy to surgery was 33, 34 and 31 days for patients with localised tumours, respectively. Patients with a metastatic tumour had surgery after a median duration of 70 and 48 days (May and June) following pre‐operative chemotherapy, respectively. In July 2020, no patients with metastatic disease were registered (Table 3).

## 4 DISCUSSION

In this study, we explored signals of the impact of the first phase of the COVID‐19 pandemic on numbers of newly diagnosed patients with a renal tumour, on tumour characteristics, presenting symptoms and on treatment delay in the first phase of the pandemic. We identified a short‐term relative effect of the pandemic on numbers of newly diagnosed patients, tumour volume and tumour stage.

In Europe, the pandemic peaked in March 2020, which was followed almost immediately by governments' imposed lockdowns. The first, most severe period of the pandemic and restrictions lasted for 3 months. In Brazil, it was suggested that the pandemic started 2 months later; however, the ICU admissions started rising only slightly later than in most European countries.^36^ The data on population mobility showed a similar pattern in duration and course as in Europe. Although political imposed restrictions varied per region in Brazil, a decline in population mobility was already observed in March 2020, indicating knowledge of the serious impact of the pandemic among the population.

During the first peak of the pandemic, we observed a relative decrease of newly diagnosed patients. This finding is consistent with reported data in other paediatric cancers^2^, ^7^, ^8^, ^38^ and other published reports support this by showing that parents and patients were afraid of catching the virus in the hospitals.^39^ However, the short decrease in newly diagnosed patients in our study might also (partly) be explained by reduced study registrations during the first COVID‐19 peak. The latter effect is anticipated to be small, as catch up data management and registration always used the date of first diagnosis by imaging. From May to July 2020, when the restrictions in Europe were eased, we observed a slight increase in the number of patients. Since the UMBRELLA study was still initiating in many countries, a general trend of increase in the number of newly diagnosed patients was observed and expected. When infection rates gradually increased again from November 2020 on, this did not seem to affect the number of new diagnoses. This was supported by patient‐based surveys, where less fear and better understanding were reported during the second wave.^40^ Soon after the first wave, caregivers promoted hospitals as a safe location and stressed the importance of treatment in children with cancer.^39^ In addition, stabilised inclusion rates may reflect adaptation of the health care system, as well as the prioritisation of care for paediatric cancer.^6^

To analyse whether the pandemic led to more adverse tumour characteristics, we assessed tumour stage and volume at presentation during the pandemic in Europe and Brazil as compared to the historical data (2005–2009). We observed that patients presented with relatively higher tumour volumes and more often with metastatic disease (Figure 1 and 2F). We were not able to assess the true impact on clinical outcomes as yet, but the higher tumour volume and the more advanced disease stage require more intensive treatment, increasing the risk of long‐term morbidity. In the months after the first lockdown, tumour volumes and stage distribution returned more or less to the historical pattern. We hypothesised that this may be explained by the fact that the small group of patients that was referred during the first lockdown consisted of patients with more severe symptoms. Therefore, we analysed route to diagnosis and presenting symptoms for patients in the different periods. For Brazil, the proportion of patients who were diagnosed based on tumour‐specific symptoms was higher in the first COVID‐19 peak (≥92% during March until July 2020), but this was also a very frequent route to diagnosis in all other time periods, compared to Europe. An increased number of symptomatic patients was not clearly present in the European countries; however, the percentage of patients with an abdominal mass and/or pain was higher in the lockdown period, which may indicate a higher burden of disease. Yet, this result was not consistent when we analysed the data for every country in Europe separately. For Brazil, we could not determine whether burden of disease was higher in the COVID‐19 period, since numbers are very small.

In Brazil, the trend of observing more patients with metastatic disease and a higher tumour volume at diagnosis was already present in March and April 2020. The decrease in the population mobility (already occurring in March and April) apparently determined the referral of more patients with metastases and a high tumour volume, rather than the peak of ICU admissions or than the more strict regulations (May–July 2020) (Figure 2B). However, the comparison with the data from the COVID‐19 period with the historical data may be hampered, since the registration of clinical data has increased tremendously since the implementation of UMBRELLA in Brazil.^41^ Therefore, there may be selection bias in the type of patients that were included in the historical cohort versus the current cohort.

The data presented here shows that children with Wilms tumour presented with a more pronounced burden of disease, but once they had reached medical services, onwards referral and treatment were not delayed. This phenomenon has been reported in some adult cancers^20^, ^21^ and is also supported by the finding that parents of children with cancer did not report major disruptions to cancer care needs.^42^ However, it has been shown that there is a difference between high income countries and low and middle income countries regarding COVID‐19‐related delay in treatment.^28^ We showed that generally, treatment (including surgery) according to the UMBRELLA protocol was feasible without much delay. Previous studies reported shortage of staff and healthcare facilities as a reason for delay in management of patients with cancer.^43^ A concern was raised that delayed presentation of localised tumours may result in a higher number of advanced disease or larger tumours in the following months of relaxation period.^44^ We did not observe such a phenomenon in our study, similar to adult reports.^45^ This may reflect the rapid efforts that have been pursued to sustain paediatric renal cancer care according to the standards, as proposed in a published guideline.^6^

### 4.1 Intercontinental experience

In general, Europe and Brazil had a similar course of the pandemic with respect to social distancing and restrictions, despite the fact that a later impact of COVID‐19 was expected in Brazil. The first case of COVID‐19 in Brazil had already been registered in February 2020 and social distancing was already advised in March, but varied largely across the different regions.^36^, ^37^ The implementation of restrictions started regionally and the content of the measures varied. Nevertheless, population mobility had already decreased in March 2020, simultaneously with lockdowns and decreased population mobilisation in Europe. This seemed to be even more influential on the adverse tumour characteristics than the incidence of SARS‐CoV‐2 infections, as reflected by the number of ICU admissions in Brazil. In Brazil, the number of newly diagnosed patients per month did not seem to decrease, in contrast to Europe.

Strengths of this study are that we present prospectively collected study data of children with Wilms tumours in Europe and Brazil. Data on tumour characteristics (stage and volume) were collected in a standardised fashion within a long‐established cooperative study group. We are confident that tumour volume has been accurately measured and registered, since the evaluation of volume reduction after pre‐operative chemotherapy are among the endpoints of the ongoing UMBRELLA study. Previously pursued, similar studies had been conducted by paediatric cancer registries with various cancers, or single‐centred experience from a regional institute.^9^, ^20^, ^32^, ^45^, ^46^ In the latter studies, influence of the pandemic on tumour characteristics, delay of diagnosis and starting treatment of the paediatric cancers could not be taken into account as specifically. In addition, the population immobilisation, apart from the restrictions, had not been included in previous studies on the influence of COVID‐19 on cancer management. Limitations in this study are that we had missing data and varying measuring methods for some parameters in the historical datasets, which may hamper comparisons. Also, due to the different durations of the compared periods (5 years in the historical cohort versus two or 3 months in the COVID‐19 period), we were limited by unequal sample sizes. Additionally, the UMBRELLA study was still initiating new national sites and the enrolment was already highly variable (and increasing) over time. Despite the merge of patients from several countries, the number of patients is too small to provide robust results for all of our study questions. The long‐term impact of the pandemic on clinical outcomes needs to be kept under review.

We conclude that the first months of the COVID‐19 pandemic led to a short‐term relative decrease in the number of newly diagnosed children with a renal tumour registered in UMBRELLA. Patients who were diagnosed during the first period of the pandemic had a relatively higher median tumour volume and more patients had metastatic disease, suggesting that only the more symptomatic cases were reaching medical attention at that time. These changes seemed to follow the severity of the COVID‐19 pandemic reflected by ICU admissions and imposed restrictions, but even more strikingly population immobilisation. Access to care seemed to be maintained, reflected by hardly any treatment delay, conceivably possible because of the high level of networking in the field of paediatric oncology. Further analysis after longer follow‐up is needed to study the influence of the impact of the COVID‐19 pandemic on long‐term clinical outcomes.

## AUTHOR CONTRIBUTIONS

**Prakriti Roy:** Conceptualization (equal); data curation (equal); formal analysis (equal); investigation (equal); visualization (equal); writing – original draft (equal); writing – review and editing (equal). **Sophie van Peer:** Data curation (equal); formal analysis (equal); investigation (equal); project administration (equal); visualization (equal); writing – original draft (equal); writing – review and editing (equal). **Rana Dandis:** Data curation (equal); formal analysis (equal); visualization (equal); writing – review and editing (equal). **Catriona Duncan:** Writing – review and editing (equal). **Joaquim Caetano de Aguirre‐Neto:** Resources (equal); writing – review and editing (equal). **Arnauld Verschuur:** Writing – review and editing (equal). **Beatriz de Camargo:** Writing – review and editing (equal). **Henrike Karim‐Kos:** Writing – review and editing (equal). **Luna Boschetti:** Writing – review and editing (equal). **Filippo Spreafico:** Writing – review and editing (equal). **Gema Lucía Ramírez Villar:** Writing – review and editing (equal). **Norbert Graf:** Resources (equal); writing – review and editing (equal). **Harm van Tinteren:** Conceptualization (equal); data curation (equal); formal analysis (equal); investigation (equal); methodology (equal); project administration (equal); resources (equal); supervision (equal); visualization (equal); writing – original draft (equal); writing – review and editing (equal). **Kathy Pritchard‐Jones:** Conceptualization (equal); investigation (equal); methodology (equal); resources (equal); supervision (equal); writing – original draft (equal); writing – review and editing (equal). **Marry M. van den Heuvel‐Eibrink:** Conceptualization (equal); formal analysis (equal); funding acquisition (equal); investigation (equal); methodology (equal); project administration (equal); resources (equal); supervision (equal); writing – original draft (equal); writing – review and editing (equal).

## CONFLICT OF INTEREST STATEMENT

The authors declare that they have no conflict of interest.

## ETHICS STATEMENT

All patients in this study provided informed consent for registration in the SIOP‐WT 2001 trial, SIOP‐RTSG 2016 UMBRELLA trial or AIEOP TW2003 study (EudraCT numbers 2007–004591‐39 and 2016–004180‐39), with ethics committee approval numbers MEC 202.134/2001/122 and MEC‐2018‐026 (Ethics Committee Erasmus Medical Center).

## Supporting information


Data S1.

## Data Availability

The data that support the findings of our study are available at the International Society of Pediatric Oncology ‐ Renal Tumor Study Group (SIOP‐RTSG) office and can be made available following standard access procedures by the corresponding author upon reasonable request.
