# Supplementary material for: Impact of the COVID‐19 pandemic on paediatric renal tumour presentation and management, a SIOP renal tumour study group study
Source: Cancer Med. 2023 Jul 26;12(16):17098–111. doi: 10.1002/cam4.6358 (PMC10501283; doi:10.1002/cam4.6358)

Supplementary material

Supplementary Table 1. Route to diagnosis and presenting symptoms pre-, during, and post-first COVID-19 wave

A. Route to diagnosis before, during and after the first COVID-19 wave

Period	Tumor specific symptoms	Non-specific assessment	Screening because of genetic predisposition	Routine check-up
**Europe**				
Pre-lockdown (April 2019-February 2020)	87 (65.9%)	29 (21.6%)	8 (6.0%)	10 (7.5%)
Lockdown (March-May 2020)	54 (69.2%)	12 (15.4%)	6 (7.7%)	6 (7.7%)
Post-lockdown (June-November 2020)	113 (64.9%)	33 (19.0%)	9 (5.2%)	19 (10.9%)
**Brazil**				
Pre-lockdown (April 2019-February 2020)	33 (86.8%)	4 (10.5%)	1 (2.6%)	0 (0%)
Period of decreased population mobility (March-April 2020)	18 (94.7%)	1 (5.3%)	0 (0%)	0 (0%)
Most severe restrictions (May-July 2020)	23 (92%)	0 (0%)	2 (8%)	0 (0%)
After lockdown (August-November 2020)	17 (85%)	3 (15%)	0 (0%)	0 (0%)

B. Presenting symptoms before, during and after the first COVID-19 wave

Period	Abdominal mass	Change in bowel habit without symptoms of acute GE	Haematuria	Hypertension	Pain	UTI	Scrotal swelling	Varicocele
**Europe**								
Pre-lockdown (April 2019-February 2020)	43 (39.3%)	8 (8.2%)	16 (16.5%)	7 (7.2%)	21 (21.6%)	2 (2.1%)	0 (0%)	0 (0%)
Lockdown (March-May 2020)	25 (49.0%)	2 (3.9%)	4 (7.8%)	4 (7.8%)	13 (25.5%)	0 (0%)	1 (2.0%)	2 (3.9%)
Post-lockdown (June - November 2020)	50 (49.0%)	5 (4.9%)	17 (16.7%)	5 (4.9%)	22 (21.6%)	3 (2.9%)	0 (0%)	0 (0%)
**Brazil**								
Pre-lockdown (April 2019-February 2020)	19 (50%)	1 (2.6%)	8 (21.1%)	0 (0%)	9 (23.7%)	1	0 (2.6%)	0 (0%)
Period of decreased population mobility (March-April 2020)	13 (68.4%)	2 (10.5%)	6 (31.6%)	2 (10.5%)	11 (57.9%)	1	0 (0%)	0 (0%)
Most severe restrictions (May-July 2020)	15 (60%)	0 (0%)	8 (32%)	3 (12%)	7 (28%)	1	0 (0%)	0 (0%)
After (August- November 2020)	14 (70%)	1 (5%)	3 (15%)	4 (20%)	8 (40%)	2	0 (0%)	0 (0%)

Calculated symptom percentages represent proportion of total symptoms reported, excluding missings. GE: gastro-enteritis; UTI: urine tract infection; NA: not available.

Supplementary Table 2. Route to diagnosis and presenting symptoms pre-, during, and post-lockdown for participating countries in Europe per country

A. The Netherlands, route to diagnosis

Period	Tumor specific symptoms	Non-specific assessment	Screening because of genetic predisposition	Routine check-up	Total
Pre-lockdown (April 2019-February 2020)	15 (71%)	3 (14%)	2 (10%)	1 (5%)	21
Lockdown (March-May 2020)	6 (60%)	3 (30%)	0	1 (10%)	10
After lockdown (June-November 2020)	8 (80%)	1 (10%)	0	1 (10%)	10

B. The Netherlands, presenting symptoms

Period	Abdominal mass	Change in bowel habit without symptoms of acute GE	Haematuria	Hyper-tension	Pain	UTI	Scrotal swelling	Varicocele	NA
Pre-lockdown (April 2019-February 2020)	14 (67%)	5 (24%)	2 (10%)	5 (24%)	8 (38%)	1 (5%)	0	0	0
Lockdown (March-May 2020)	6 (60%)	2 (20%)	0	2 (20%)	2 (20%)	0	0	0	0
After lockdown (June-November 2020)	7 (70%)	1 (10%)	1 (10%)	3 (30%)	3 (30%)	0	0	0	0

GE: gastro-enteritis; UTI: urine tract infection; NA: Not available.

C. United Kingdom, route to diagnosis

Period	Tumor specific symptoms	Non-specific assessment	Screening because of genetic predisposition	Routine check-up	Total
Pre-lockdown (April 2019-February 2020)	17 (74%)	5 (22%)	1 (4%)	NA	23
Lockdown (March-May 2020)	11 (85%)	2 (15%)	0	NA	13
After lockdown (June-November 2020)	18 (86%)	1 (5%)	2 (10%)	NA	21

NA: not applicable.

D. United Kingdom, presenting symptoms

Period	Abdominal mass	Change in bowel habit without symptoms of acute GE	Haematuria	Hyper-tension	Pain	UTI	Scrotal swelling	Varicocele	NA
Pre-lockdown (April 2019-February 2020)	9 (39%)	2 (9%)	4 (17%)	0	6 (26%)	0	0	0	1 (4%)
Lockdown (March-May 2020)	6 (46%)	1 (8%)	0	0	5 (38%)	0	1 (8%)	0	2 (15%)
After lockdown (June-November 2020)	11 (52%)	0	5 (24%)	2 (10%)	1 (5%)	1 (5%)	0	0	4 (19%)

GE: gastro-enteritis; UTI: urine tract infection; NA: Not available.

E. Spain, route to diagnosis

Period	Tumor specific symptoms	Non-specific assessment	Screening because of genetic predisposition	Routine check-up	Total
Pre-lockdown (April 2019-February 2020)	11 (52%)	3 (14%)	3 (14%)	4 (19%)	21
Lockdown (March-May 2020)	3 (60%)	0	1 (20%)	1 (20%)	5
After lockdown (June-November 2020)	11 (55%)	4 (20%)	0	5 (25%)	20

F. Spain, presenting symptoms

Period	Abdominal mass	Change in bowel habit without symptoms of acute GE	Haematuria	Hyper-tension	Pain	UTI	Scrotal swelling	Varicocele	NA
Pre-lockdown (April 2019-February 2020)	4 (19%)	0	1 (5%)	1 (5%)	3 (14%)	0	0	0	3 (14%)
Lockdown (March-May 2020)	2 (40%)	0	0	0	1 (20%)	0	0	0	2 (40%)
After lockdown (June-November 2020)	5 (25%)	0	1 (5%)	0	3 (15%)	1 (5%)	0	0	4 (20%)

GE: gastro-enteritis; UTI: urine tract infection; NA: Not available.

G. Italy, route to diagnosis

Period	Tumor specific symptoms	Non-specific assessment	Screening because of genetic predisposition	Routine check-up	Total
Pre-lockdown (April 2019-February 2020)	22 (56%)	11 (28%)	1 (3%)	5 (13%)	39
Lockdown (March-May 2020)	7 (64%)	3 (27%)	0	1 (9%)	11
After lockdown (June-November 2020)	14 (52%)	9 (33%)	0	4 (15%)	27

H. Italy, presenting symptoms

Period	Abdominal mass	Change in bowel habit without symptoms of acute GE	Haematuria	Hypertension	Pain	UTI	Scrotal swelling	Varicocele	NA
Pre-lockdown (April 2019-February 2020)	14 (36%)	0	6 (15%)	1 (3%)	3 (8%)	1 93%)	0	0	2 (51%)
Lockdown (March-May 2020)	6 (55%)	0	1 (9%)	0	2 (18%)	0	0	1 (9%)	0
After lockdown (June-November 2020)	7 (26%)	0	4 (15%)	0	3 (11%)	0	0	0	0

GE: gastro-enteritis; UTI: urine tract infection; NA: Not available.

I. France, route to diagnosis

Period	Tumor specific symptoms	Non-specific assessment	Screening because of genetic predisposition	Routine check-up	Total
Pre-lockdown (April 2019-February 2020)	3 (100%)	0	0	0	3
Lockdown (March-May 2020)	5 (42%)	3 (25%)	1 (8%)	3 (25%)	12
After lockdown (June-November 2020)	19 (53%)	12 (33%)	1 (3%)	3 (+1 prenatal dx) (11%)	36

J. France, presenting symptoms

Period	Abdominal mass	Change in bowel habit without symptoms of acute GE	Haematuria	Hypertension	Pain	UTI	Scrotal swelling	Varicocele	NA
Pre-lockdown (April 2019-February 2020)	2 (67%)	0	1 (33%)	0	1 (33%)	0	0	0	0
Lockdown (March-May 2020)	3 (25%)	0	1 (8%)	1 (8%)	2 (6%)	0	0	1 (8%)	1 (8%)
After lockdown (June-November 2020)	9 (25%)	0	5 (14%)	0	4 (11%)	0	0	0	1 (3%)

GE: gastro-enteritis; UTI: urine tract infection; NA: Not available.

K. Germany, route to diagnosis

Period	Tumor specific symptoms	Non-specific assessment	Screening because of genetic predisposition	Routine check-up	Total
Pre-lockdown (April 2019-February 2020)	20	6	1	0	26
Lockdown (March-May 2020)	22	1	4	0	27
After lockdown (June-November 2020)	44	5	6	5	65

L. Germany, presenting symptoms

Period	Abdominal mass	Change in bowel habit without symptoms of acute GE	Haematuria	Hypertension	Pain	UTI	Scrotal swelling	Varicocele	NA
Pre-lockdown (April 2019-February 2020)	1	1	2	0	0	0	0	0	16
Lockdown (March-May 2020)	2	0	2	0	1	0	0	0	17
After lockdown (June-November 2020)	7	3	7	0	4	1	0	0	27

GE: gastro-enteritis; UTI: urine tract infection; NA: Not available.

Supplementary Figure 1. Monthly number of patients diagnosed with a new renal tumour during the pandemic and the average monthly number of patients with a new renal tumour in the historical cohort (2005-2009) as per country

A. The Netherlands

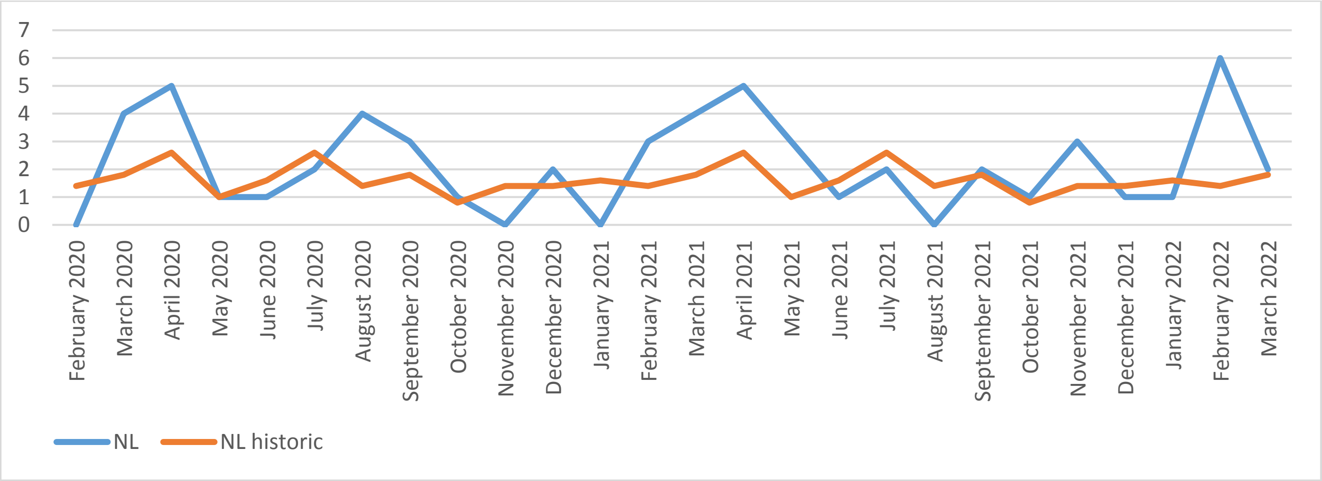

B. United Kingdom

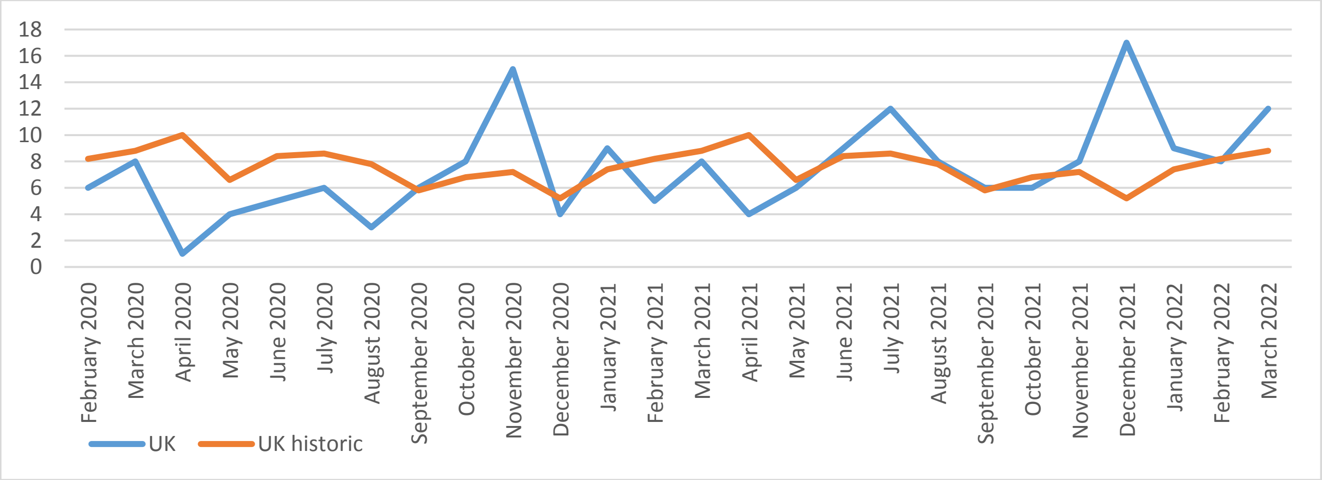

C. Spain

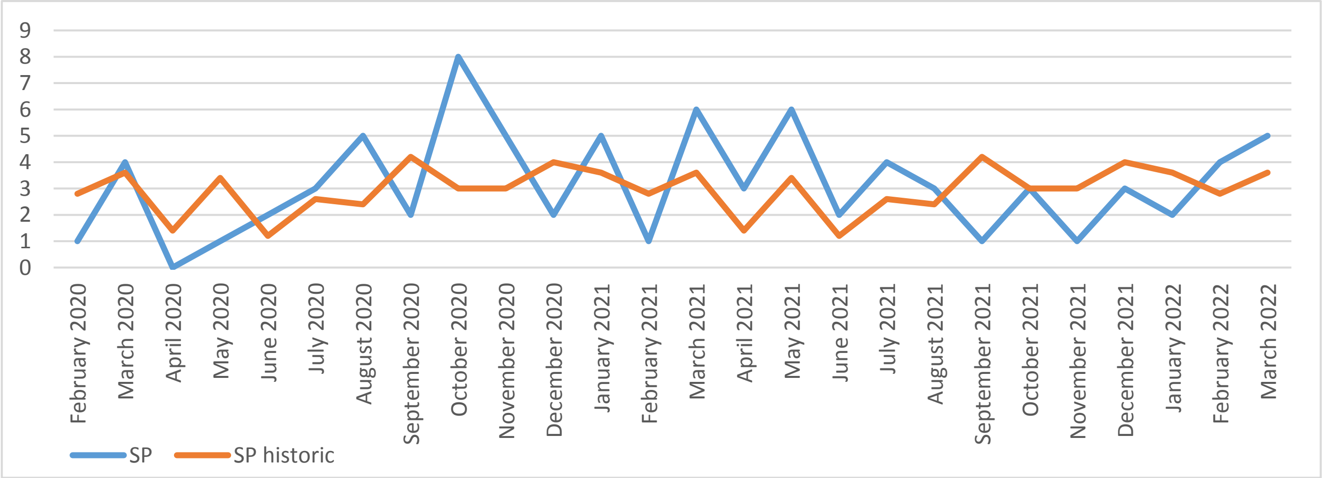

D. Italy

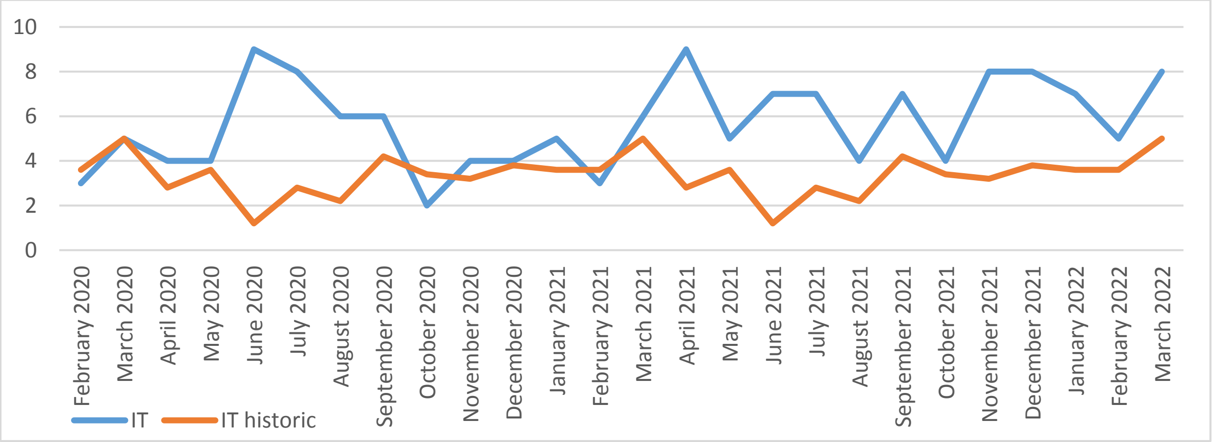

E. Germany

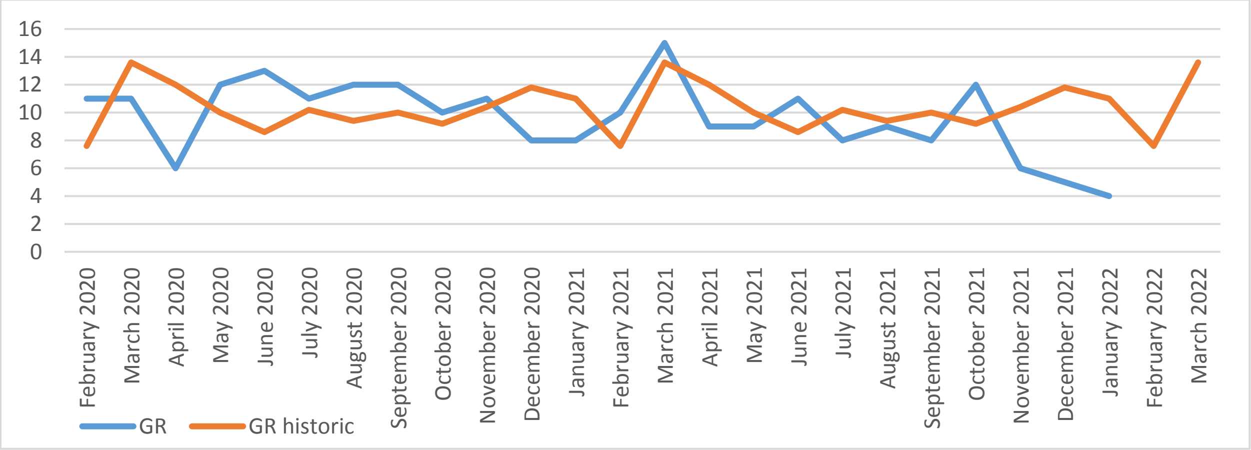

F. France

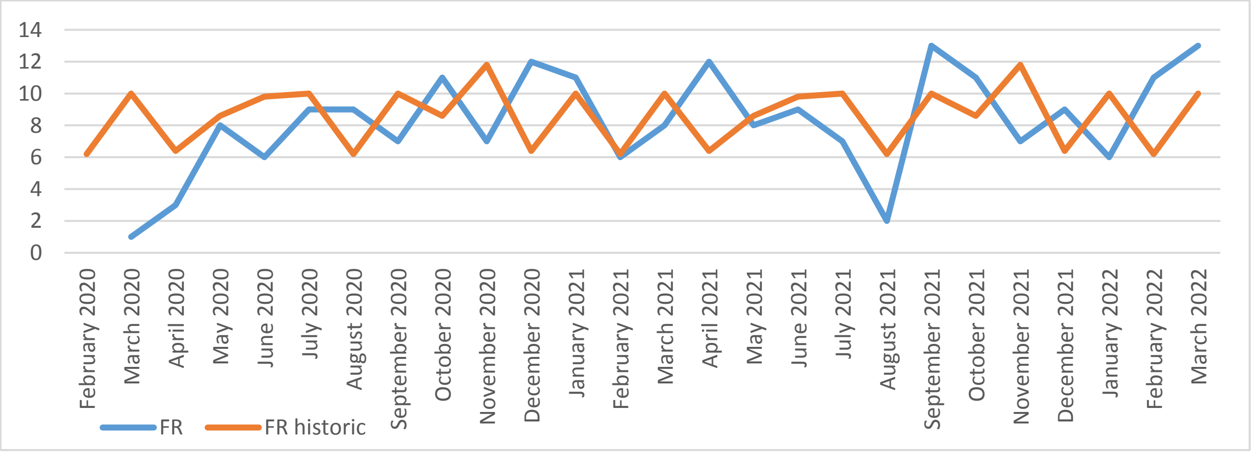

Supplementary Figure 2

A. Route to diagnosis before, during and after lockdown in Europe

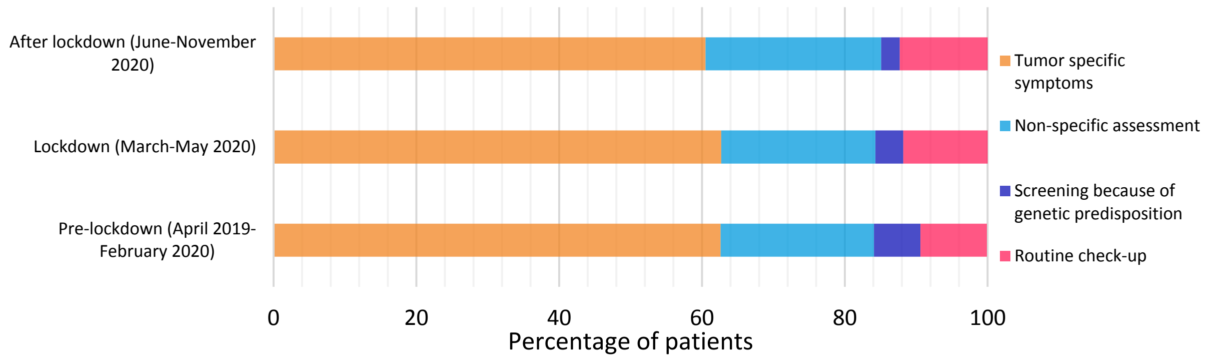

B. Presenting symptoms before, during and after lockdown in Europe

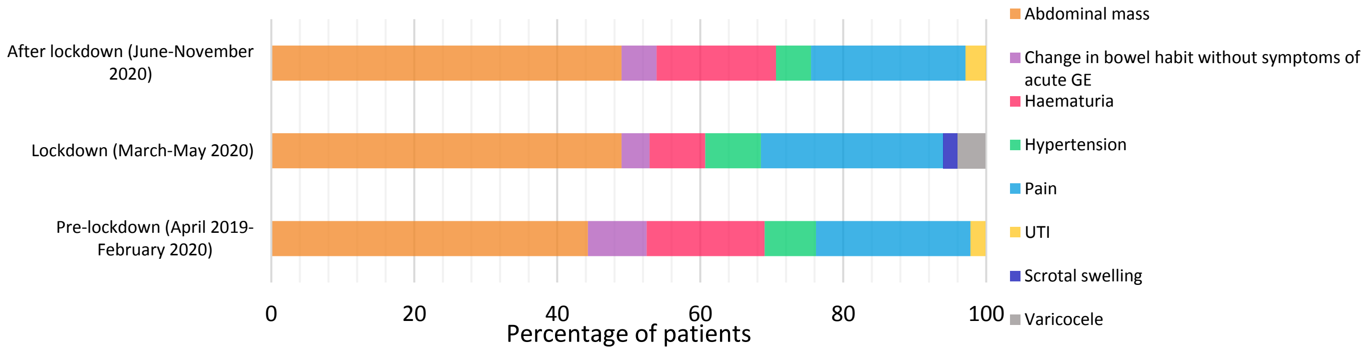

Supplementary Figure 3

A. Route to diagnosis before, during and after first COVID-19 peak in Brazil

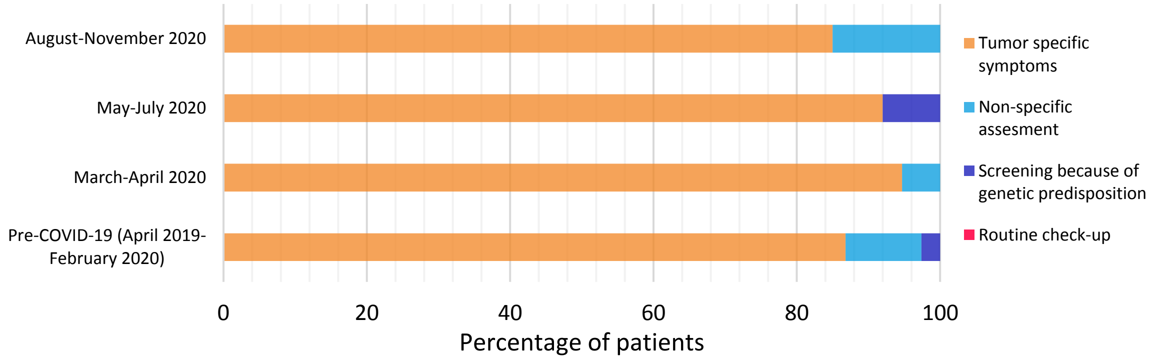

B. Presenting symptoms before, during and after first COVID-19 peak in Brazil

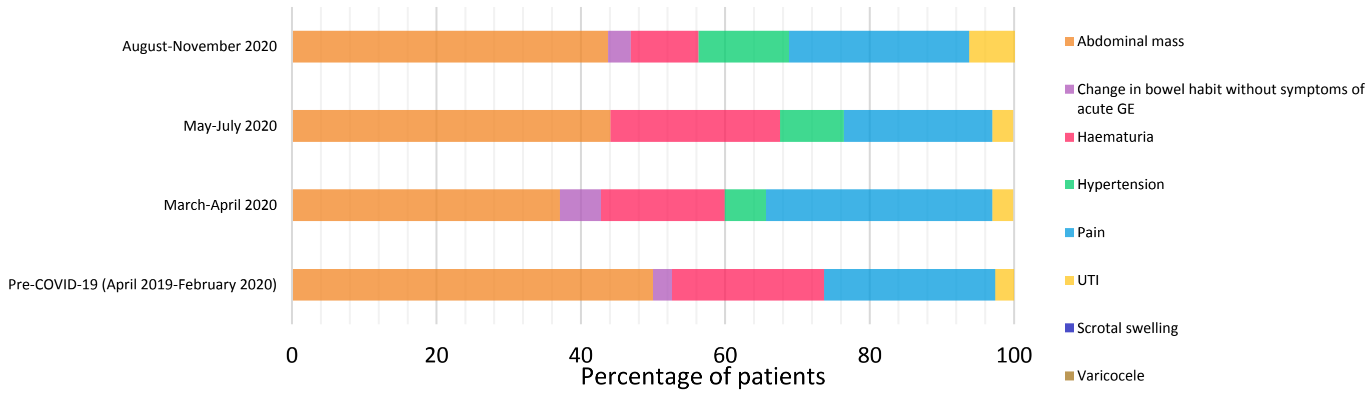

Supplement: Supplementary file 1 — Data S1. [file CAM4-12-17098-s001.pdf]
